# Screening biotoxin aptamer and their application of optical aptasensor in food stuff: a review

**DOI:** 10.3389/fchem.2024.1425774

**Published:** 2024-07-24

**Authors:** Jiefang Sun, Meng Zhang, Qianlong Gao, Bing Shao

**Affiliations:** ^1^ Beijing Center for Disease Prevention and Control, Beijing, China; ^2^ School of Public Health, Capital Medical University, Beijing, China; ^3^ School of Chinese Medicine, Yunnan University of Traditional Chinese Medicine, Kunming, China

**Keywords:** biotoxins, aptamers, food safety, optical, biosensors

## Abstract

Biotoxins are ranges of toxic substances produced by animals, plants, and microorganisms, which could contaminate foods during their production, processing, transportation, or storage, thus leading to foodborne illness, even food terrorism. Therefore, proposing simple, rapid, and effective detection methods for ensuring food free from biotoxin contamination shows a highly realistic demand. Aptamers are single-stranded oligonucleotides obtained from the systematic evolution of ligands by performing exponential enrichment (SELEX). They can specifically bind to wide ranges of targets with high affinity; thus, they have become important recognizing units in safety monitoring in food control and anti-terrorism. In this paper, we reviewed the technical points and difficulties of typical aptamer screening processes for biotoxins. For promoting the understanding of food control in the food supply chain, the latest progresses in rapid optical detection of biotoxins based on aptamers were summarized. In the end, we outlined some challenges and prospects in this field. We hope this paper could stimulate widespread interest in developing advanced sensing systems for ensuring food safety.

## 1 Introduction

Biotoxins, also known as natural toxins, are groups of inherently small molecules, peptides, or proteins, which are produced during the metabolism of plants, microorganisms, and animals (Montecucco, 2012). Food contamination by biotoxin usually occurs due to unsterilized processing, improper storage, as well as microbial spoilage, whereas others are inherent ingredients in plants, or might be produced by environmental stimuli, such as shellfish toxin (Rather et al., 2017). Biotoxins are prevalent in food-poisoning incidents around the world and are the main culprits of various acute and chronic human diseases. According to the World Health Organization (WHO), the consumption of mycotoxin-contaminated grains (Alshannaq and Yu, 2017), meats, and vegetables (especially fermented foods) with poor sanitation and bacterial contamination has been the first cause of foodborne illness (WHO, 2015). Aquatic and marine foods such as shellfish, fish, and even water are contaminated with algal biotoxins (Oliveira et al., 2011), as well as the normal metabolites produced by some plants for natural defenses and resistance; all of these become toxins in other organisms (Fletcher and Netzel, 2020). According to the classification of toxin-producing organisms, they are classified as animal toxins (snake venom and bee venom), plant toxins (lectins and alkaloids), microbial toxins (bacteria and molds), and marine biotoxins (algae and shellfish) (Picardo et al., 2020). Specifically, some highly toxic biotoxins, such as ricin, acacia toxin, and tetrodotoxin, have been listed in the Organization for the Prohibition of Chemical Weapons (OPCW) and have a risk of being used by terrorist organizations to threaten public security (Anderson, 2012). Biotoxins act on the cell membrane or ribosomal protein with high specificity, resulting in varying degrees of toxicity, which are not only harmful to the health of consumers (Rather et al., 2017) but also cause huge economic losses to the planting and aquaculture industries. Although biotoxins cannot replicate themselves, they are generally chemically stable and highly toxic. They can persist for a long time after an organism dies, further entering food chains, and finally triggering food safety and public health crises (Banach et al., 2020). In the past, due to the lack in knowledge and reliable detecting methods for biotoxins, food safety verification had been neglected, thus leading to thousands of foodborne poisoning events. With the continuous improvement of the economic level, people’s health awareness also improved significantly. Therefore, highly sensitive and rapid methods for detecting biotoxins are urgently required to ensure foods free from biotoxin contamination (Bacha et al., 2023).

Until now, various analytical tools have been proposed to determine biotoxin presence in foods, which can be mainly divided into laboratory precision detection based on high-performance liquid chromatography/mass spectrometry (HPLC–MS) (Ahuja et al., 2023) and on-site rapid screening represented by affinity-recognition sensors (Song et al., 2019; Sarkar et al., 2023). Between them, LC–MS-based methods allow precious, high throughput, and ultra-sensitive detection of biotoxins. Benefited by the development of high-resolution mass spectrometry (HRMS) and tandem mass spectrometry (MS/MS), the sensitivity of biotoxin detection could further reach to ultra-low levels, making them the gold standard for the identification and quantification of toxins in food by research workers, regulatory agencies, and the food industry. However, LC–MS-based methods generally involve complicated sample preparation, costly instruments, and well-trained personnel; these features confine their applications in daily monitoring. In contrast, on-site rapid screening biosensors, which are typically composed of highly specific recognition ligands (e.g., antibodies and aptamers) with advanced signal reporting materials, could well meet the need of the modern society for daily safety monitoring of food samples. With its rapid response, ease of use, and affordable advantages, it has become an indispensable and powerful tool to ensure the safety and quality of the food supply chain (Howes et al., 2014).

**SCHEME 1:**
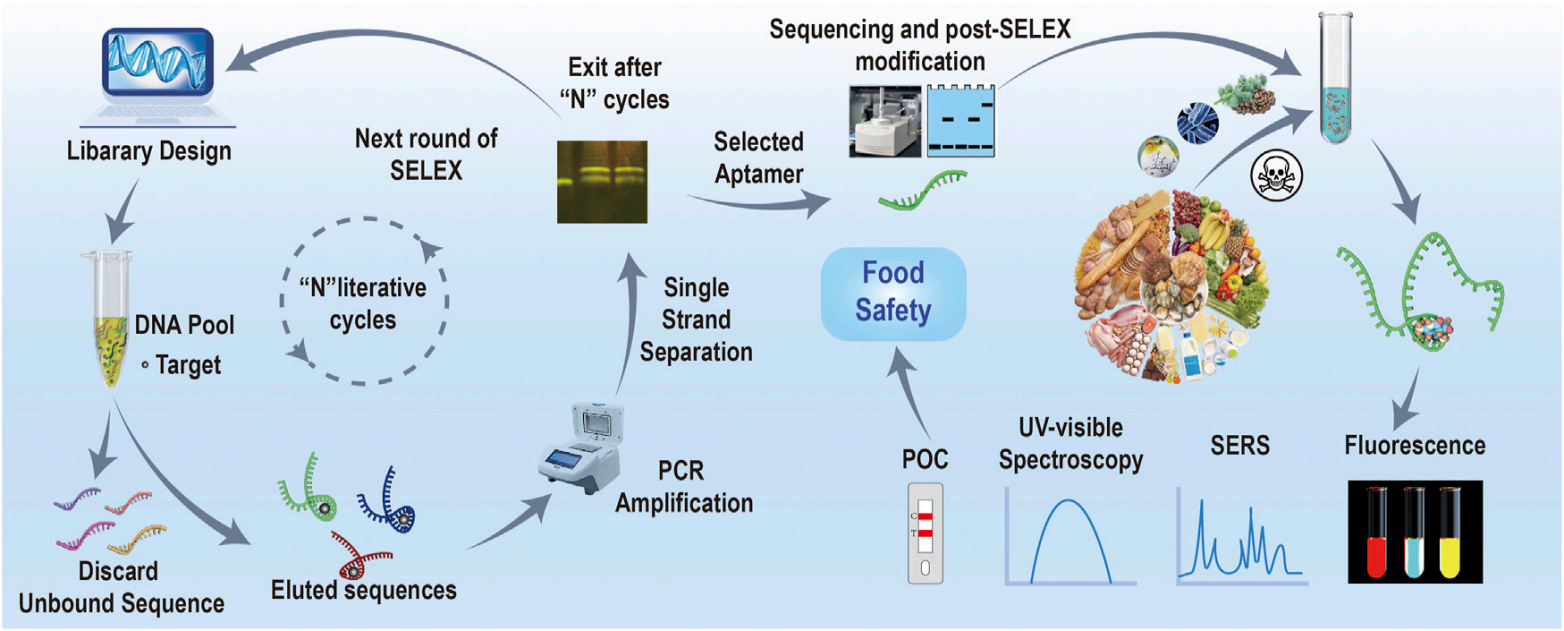
Screening aptamer for biotoxin by SELEX and their food safety application.

Aptamers are single-stranded DNA or RNA fragments of between 10 and 100 bases obtained from artistically synthesized libraries (Scheme 1). They show unique three-dimensional structures, which could form helices and single-stranded loop-like structures to match specific targets (small molecules, proteins, cells, *etc*.) through the non-covalent bonds, including hydrogen bonding, van der Waals forces, electrostatic interactions, and π–π* stacking interactions. Aptamers have multiple advantages of good stability, easy chemical modification, high affinity and specificity, as well as low immunogenicity. Until now, dozens of aptamers of biotoxins have been obtained by the Systematic Evolution of Ligands by Exponential Enrichment (SELEX) technology, as listed in Table 1. Aptamers have also widely been used for constructing various biotoxin-specific biosensors (He et al., 2023). In this field, optical biosensors based on aptamer recognition (optical aptasensors) have gained more scientific attention and offer promising applications in the field of food safety and detection. They could transfer biorecognition events into quantifiable optical signals without being restricted by the environment and equipment, and they are rapid, accurate, cheap, and portable. Specifically, through integrating aptamers as signal reporting units with excellent optical materials (Loyez et al., 2022), such as upconversion luminescent NPs, colloidal gold NPs, and time-resolved fluorescent NPs, the detecting performance of aptasensors could further be improved (Liu B. et al., 2022).

**TABLE 1:** Detailed information about the aptamers selected for mycotoxins.

Num	Biotoxin	Sequence (5′–3′)	SELEX method	Selection buffer	K_d_	Ref.
1	AFB_1_	GTT​GGG​CAC​GTG​TTG​TCT​CTC​TGT​GTC​TCG​TGC​CCT​TCG​CTA​GGC​CCA​CA	Resin SELEX	10 mM HEPES, 120 mM NaCl, 5 mM KCl, 5 mM MgCl_2_, pH 7.0	60 nM	Shkembi et al. (2021)
2	AFB_1_	AGC​AGC​ACA​GAG​GTC​AGA​TGG​TGC​TAT​CAT​GCG​CTC​AAT​GGG​AGA​CTT​TAG​CTG​CCC​CCA​CCT​ATG​CGT​GCT​ACC​GTG​AA	Magnetic bead-SELEX	100 mM NaC1, 20 mM Tris-HCl, 2 mM MgCl_2_, 5 mM KCl, 1 mM CaCl_2_, 0.02% Tween-20, pH 7.0	11.39 nM	Ma et al. (2014)
3	AFB_2_	AGC​AGC​ACA​GAG​GTC​AGA​TGC​TGA​CAC​CCT​GGA​CCT​TGG​GAT​TCC​GGA​AGT​TTT​CCG​GTA​CCT​ATG​CGT​GCT​ACC​GTG​AA	Magnetic bead-SELEX	100 mM NaCl, 20 mM Tris-HCl, 2 mM MgCl_2_, 5 mM KCl, 1 mM CaCl_2_, 0.02% Tween-20, pH 7.0	9.83 nM	Ma et al. (2015)
4	AFM_1_	ATC​CGT​CAC​ACC​TGC​TCT​GAC​GCT​GGG​GTC​GAC​CCG​GAG​AAA​TGC​ATT​CCC​CTG​TGG​TGT​TGG​CTC​CCG​TAT	Magnetic bead-SELEX	20 mM Tris-HCl, 100 mM NaCl, 2 mM MgCl_2_, 5 mM KCl, 1 mM CaCl_2_, pH 7.6	35 nM	Malhotra et al. (2014)
5	OTA	GAT​CGG​GTG​TGG​GTG​GCG​TAA​AGG​GAG​CAT​CGG​ACA	Resin-SELEX	10 mM HEPES, 120 mM NaCl, 5 mM KCl, 5 mM MgCl_2_, pH 7.0	50 nM	Cruz-Aguado and Penner (2008)
6	ZEN	AGC​AGC​ACA​GAG​GTC​AGA​TGT​CAT​CTA​TCT​ATG​GTA​CAT​TAC​TAT​CTG​TAA​TGT​GAT​ATG​CCT​ATG​CGT​GCT​ACC​GTG​AA	Magnetic bead-SELEX	100 mM NaCl, 20 mM Tris-HCl,2 mM MgCl_2_, 5 mM KCl,1 mM CaCl_2_, 0.02% Tween-20, pH 7.4	41 nM	Chen et al. (2013)
7	FB_1_	ATACCAGCTTATTCAATTAATCGCATTACCTTATACCAGCTTATTCAATTACGTCTGCACAIACCAGCTTATTCAATTAGATAGTAAGTGCAATCT	Magnetic bead-SELEX	100 mM NaCl, 20 mM Tris, 2 mM MgCl, 5 mM KCl, 1 mM CaCl_2_, pH7.6	100 nM	McKeague et al. (2010)
8	T-2	AGC​TCA​GAA​GCT​TGA​TCC​TGT​ATA​TCA​AGC​ATC​GCG​TGT​TTA​CAC​ATG​CGA​GAG​GTG​AAG​ACT​CGA​AGT​CGT​GCA​TCT​G	Graphene oxide-SELEX	10 mM Tris-HCl, 150 mM NaCl, 10 mM KCl, 2.5 mM MgCl_2_, pH 7.4	20.8 nM	Chen et al. (2014)
9	DON	GCA​TCA​CTA​CAG​TCA​TTA​CGC​ATC​GTA​GGG​GGG​ATC​GTT​AAG​GAA​GTG​CCC​GGA​GGC​GGT​ATC​GTG​TGA​AGT​GCT​GTC​CC	Magnetic bead-SELEX	50 mM Tris-HCl, 5 mM KCl, 100 mM NaCl, 1 mM MgCl_2_, pH 7.4	N/A	Patent:CN-102559686-A (2024)
10	DON	GGC​ACG​GAG​TCT​GCC​CGA​CTG​GGG​ACC​CTA​GGA​TCA​CTT​A	Sepharose bead-SELEX	2 mM KH_2_PO_4_, 8 mM Na_2_HPO_4_, 136 mM NaCl, 2.6 mM KC1, 5 mM MgCl_2_, 1 μg mL^-1^ tRNA, 0.02% Tween-20	40.5 nM	Patent:CN-113774063-A (2024)
11	Patulin	CGA​AAT​CGC​GTC​CAG​TGT​TGG​GGC​GTG​CTT​ATC​CTT​ACA​CGA​TTT​ACC​TGA​AAC​GCA​CCG​TAC​TGA​ACT​ACG​GCG​AGG​TC	Biolayer interferometry-SELEX	50 mM Tris HCl, pH 8	82 nM	Mukherjee et al. (2022)
12	STX	TAG​GGA​AGA​GAA​GGA​CAT​ATG​ATG​GCA​CAA​GGC​CTC​ATC​AAT​CGG​TAT​ACG​GGT​TGA​CTA​GTA​CAT​GAC​CAC​TTG​A	Graphene oxide-SELEX	2.7 mM KCl, 140 mM NaCl, pH 7.4	50.8 nM	Ha et al. (2019)
13	STX	ATAGGAGTCACGACGACCAGCTTTTTACAAAATTCTCTTTTTACCTAIATTATGAACAGATATGTGCGTCTACCTCTTGA	Magnetic bead-SELEX	2.7 mM KCl, 140 mM NaCl, 0.05% Tween-20, pH 7.4	61.4 nM	Yu et al. (2021)
14	STX	GGT​ATT​GAG​GGT​CGC​ATC​CCG​TGG​AAC​ATG​TTC​ATT​GGG​CGC​ACT​CCG​CTT​TCT​GTA​GAT​GGC​TCT​AAC​TCT​CCT​CT	Magnetic bead-SELEX	2.7 mM KCl, 140 mM NaCl, 0.05% Tween-20, pH 7.4	3,840 nM	Handy et al. (2013)
15	OA	GGT​CAC​CAA​CAA​CAG​GGA​GCG​CTA​CGC​GAA​GGG​TCA​ATG​TGA​CGT​CAT​GCG​GAT​GTG​TGG	Agarose bead-SELEX	50 mM Tris, pH 7.5, 150 mM NaC1, 2 mM MgCl_2_, pH 7.5	77 nM	Eissa et al. (2013)
16	OA	ATT​TGA​CCA​TGT​CGA​GGG​AGA​CGC​GCA​GTC​GCT​ACC​ACC​T	Graphene oxide-SELEX	50 mM Tris, 150 mM NaCl, 2 mM MgCl_2_, pH 7.4	40 nM	Gu et al. (2016)
17	PTX	GGA​GGT​GGT​GGG​GAC​TTT​GCT​TGT​ACT​GGG​CGC​CCG​GTT​GAA	Magnetic bead-SELEX	20 mM Tris, 100 mM NaCl, 2 mM MgCl_2_, 5 mM KCl, pH 7.5	84.3 nM	Gao et al. (2017)
18	TTX	ATA​GGA​GTC​ACG​ACG​ACC​AGT​CAA​ATT​TTC​GTC​TAC​TCA​ATC​TTT​CTG​TCT​TAT​CTA​TGT​GCG​TCT​ACC​TCT​TGA	Magnetic bead-SELEX	2.7 mM KCl, 140 mM NaCl, 0.05% Tween-20, pH 7.4	44.1 nM	Yu et al. (2021)
19	DTX-1	CCA​CCA​GGC​CAA​ACA​CGA​CCC​CAA​ACA	Magnetic bead-SELEX	50 mM Tris-HCl, 150 mM NaCl, 2 mM MgCl_2_, pH 7.5	64 nM	Li et al. (2020)
20	BTX-2	GGC​CAC​CAA​ACC​ACA​CCG​TCG​CAA​CCC​CGA​GAA​CCG​AAG​TAG​TGA​TCA​TGT​CCC​TGC​GTG	Divinyl sulfone bead-SELEX	50 mM Tris, 10 mM MgCl_2_, pH 7.5	92 nM	Eissa et al. (2015)
21	BTX-2	GAG​GCA​GCA​CTT​CAC​ACG​ATC​TGT​GAA​GTT​TTT​GTC​ATG​GTT​TGG​GGG​TGG​TAG​GTA​ATG​ACT​GTA​GAG​ATG	Microwell-SELEX	20 mM HEPES, 120 mM, NaCl, 5 mM KCl, 1 mM CaCl_2_, 1 mM MgCl_2_	4.8 µM	Tian et al. (2016)
22	GTX 1/4	AAC​CTT​TGG​TCG​GGC​AAG​GTA​GGT​T	Graphene oxide-SELEX	20 mM Tris-HCl, 10 mM MgCl_2_, pH 7.5	17.7 nM	Gao et al. (2016)
23	DA	ATAGGAGTCACGACGACCAGAAAAAIAATTTAAATTTTCTACCCAATGCTTTTCGCATAATATGTGCGTCTACCTCTTGA	Magnetic bead-SELEX	2.7 mM KCl, 140 mM NaCl, 0.05% Tween-20, pH 7.4	62.1 nM	Yu et al. (2021)
24	MC-LR	GGC​GCC​AAA​CAG​GAC​CAC​CAT​GAC​AAT​TAC​CCA​TAC​CAC​CTC​ATT​ATG​CCC​CAT​CTC​CGC	Sepharose bead-SELEX	50 mM Tris, 150 mM NaCl, 2 mM MgCl_2_, pH 7.5	50 nM	Ng et al. (2012)
25	MC-YR	CAC​GCA​ACA​ACA​CAA​CAT​GCC​CAG​CGC​CTG​GAA​CAT​ATC​CTA​TGA​GTT​AGT​CCG​CCC​ACA	Sepharose bead-SELEX	50 mM Tris, 150 mM NaCl, 2 mM MgCl_2_, pH 7.5	28 nM	Ng et al. (2012)
26	MC-LA	GGA​CAA​CAT​AGG​AAA​AAG​GCT​CTG​CTA​CCG​GAT​CCC​TGT​TGT​ATG​GGC​ATA​TCT​GTT​GAT	Sepharose bead-SELEX	50 mM Tris, 150 mM NaCl, 2 mM MgCl_2_, pH 7.5	193 nM	Ng et al. (2012)
27	SEB	GGT​ATT​GAG​GGT​CGC​ATC​CAC​TGG​TCG​TTG​TTG​TCT​GTT​GTC​TGT​TAT​GTT​GTT​TCG​TGA​TGG​CTC​TAA​CTC​TCC​TCT	Magnetic bead-SELEX	2.7 mM KCl, 140 mM NaCl, 0.05% Tween-20, pH 7.4	N/A	DeGrasse (2012)
28	SEA	AGG​CGA​TTA​CGC​TTC​TTG​TAC​TTC​AAT​AAC​GAC​TCA​ACT​C	Magnetic bead-SELEX	20 mM Tris, 100 mM NaCl, 5 mM KCl, 1 mM CaCl_2_, 1 mM MgCl_2_, pH 7.4	48.57 nM	Huang et al. (2014)
29	SEC_1_	AGC​AGC​ACA​GAG​GTC​AGA​TGT​ATA​CTT​CTA​AAA​TTT​GTT​TGT​ATC​TAC​GAT​GTT​CTT​CGT​CCT​ATG​CGT​GCT​ACC​GTG​AA	Graphene oxide-SELEX	20 mM Tris, 100 mM NaCl, 5 mM KCl, 1 mM CaCl_2_, 1 mM MgCl_2_, pH 7.4	65.14 nM	Huang et al. (2015)
30	Shiga toxin1	ATC​CAG​AGT​GAC​GCA​GCA​GTA​GTT​TGT​TGG​TTA​TTA​CGG​CGG​GTT​GCG​ATG​GGT​GCG​AAT​CGG​TGG​ACA​CGG​TGG​CTT​AGT	Biolayer interferometry-SELEX	10 mM phosphate buffer, 100 mM NaCl, 2.5 mM KCl, 5 mM MgCl_2_, pH 7.2	47.2 p.m.	Kaur et al. (2020)
31	Shiga toxin2	ATC​CAG​AGT​GAC​GCA​GCA​GGA​AAG​GAC​GTC​AAA​TTA​GGG​GCG​GGA​CAA​CGA​AAG​CCC​ACA​ACT​GGA​CAC​GGT​GGC​TTA​GT	Biolayer interferometry-SELEX	10 mM phosphate buffer, 100 mM NaCl, 2.5 mM KCl, 5 mM MgCl2, pH 7.2	28.6 p.m.	Kaur et al. (2020)
32	Botulinum neurotoxin	ATA​CCA​GCT​TAT​TCA​ATT​GAC​ATG​ACT​GGG​ATT​TTT​GGC​GAA​ATC​GAA​GGA​AGC​GGA​GAG​ATA​GTA​AGT​GCA​ATC​T	Agarose bead-SELEX	20 mM HEPES, 150 mM NaCl; 5 mM KCl, 2 mM MgCl2, 2 mM CaCl2, pH 7.4	3 nM	Tok and Fischer (2008)

In this paper, a concise description of aptamer screening processes for typical biotoxins and the key technical challenges were briefly introduced. Then, the source and characteristics of different biotoxins were summarized, and the general design for optical aptasensors using advanced materials and the nucleic acid isothermal amplification strategy were introduced in each chapter comprehensively. Finally, the challenges and directions of future research of the existing rapid detection methods were discussed. We hoped that this review would inspire research workers to develop more optical aptasensors with superior performance for ensuring food safety.

## 2 Screening aptamers for biotoxins and their challenges

Nucleic acid aptamers are isolated by the Systematic Evolution of Ligands by EXponential enrichment (SELEX) screening technique, which was first proposed by Tuerk and Gold (1990). Using this technique, aptamers can be screened from a library of random single-stranded nucleic acid with high target affinity (Radom et al., 2013). Generally, SELEX for discovering biotoxin aptamers mainly involves four steps: 1. Oligonucleotide library design and optimization. Random nucleic acid libraries are usually obtained by chemical synthesis but can also be constructed by genomic DNA design and *in vitro* transcription (Pan and Clawson, 2010). 2. Improvement of screening methods: currently, dozens of SELEX methods and their derived strategies have been reported by different groups worldwide, including immobilized-based, non-immobilized-based, and assisted SELEX strategy for small-molecule targets (Abdelsayed et al., 2017; Yang et al., 2019). 3. Complex separation: the complex between the targets and their oligonucleotides on the surface of solid interfaces can be separated from the unbound ones, and then they directly undergo PCR amplified without elution (Stoltenburg et al., 2005). 4. Sub-library enrichment: after acquiring aptamers from the oligonucleotide library, single-strand splitting or truncation for structure optimization would be conducted subsequently for improving their affinities and specificities (Bawazer et al., 2014; Gu et al., 2018).

**FIGURE 1:**
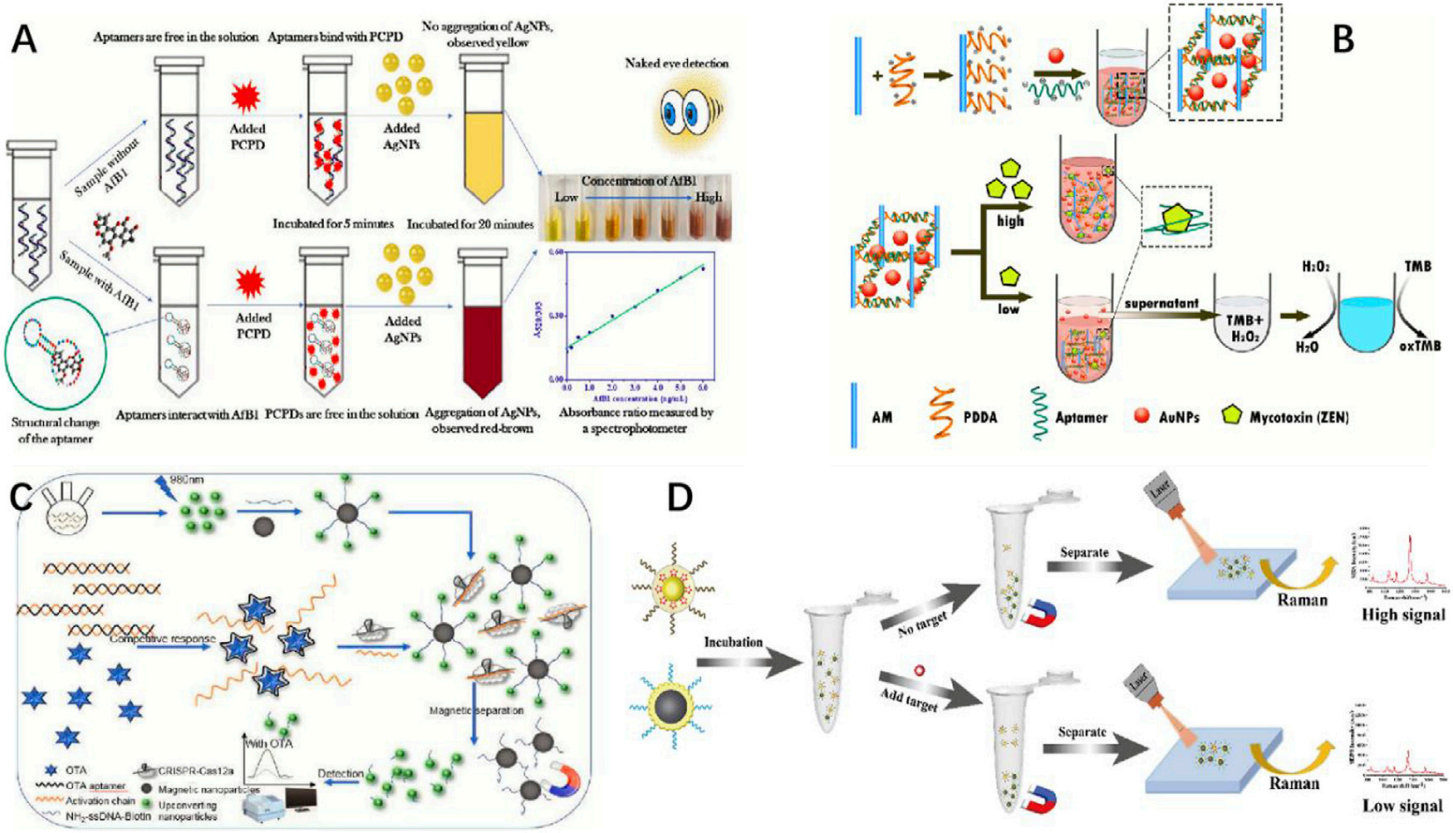
**(A)** Schematic demonstration of AfB1 detection based on colorimetric aptasensor utilizing the specific aptamer, PCPD, and AgNPs (Yu et al., 2021). **(B)** Schematic diagram of the AuNPs encapsulated PDDA-aptamer hydrogel for ultrasensitive colorimetry of ZEN (Lyu et al., 2021). **(C)** Schematic showing upconversion-mediated CRISPR-Cas12a biosensor that sensitively detects OTA (Niazi et al., 2022). **(D)** Schematic representation of the universal surface-enhanced Raman scattering (SERS) aptasensor platform for trace detection (Li et al., 2019).

Developing a rational screening strategy is the footstone to obtaining well-performed aptamers (Yu et al., 2021) (Figure 1). Although standard screening processes for biotoxins have not been proposed at the present stage, some basic evaluating rules for enhancing the screening success rate are widely accepted currently (McKeague et al., 2015). First, biotoxins of larger molecular weights, fewer rotational bonds, and more aromatic moieties could be more suitable for SELEX because they contain more potential binding interfaces and sites, a lower entropic binding retardation, and potential π–π* stacking effect with aptamer bases. Second, the low polarity and poor water solubility of some biotoxins make them incompatible with traditional water phase conducted screenings. Third, biotoxins require specialized conjugation chemistry or complicated chemical synthesis steps to achieve immobilization, which causes challenges and complexities for SELEX. In the process of aptamer screening, it is usually necessary to immobilize the targets on certain solid phase carriers, which could be magnetic beads, graphene, gold nanoparticles (AuNPs), microcolumns, chips, *etc*., to separate nonbinding sequences with the complex (Lyu et al., 2021), which inevitably involve molecular structure derivation and chemical coupling processes. However, biotoxins have far fewer functional groups and simple chemical structures. Any alterations may result in fundamental molecular changes, changing the physicochemical properties inherently, which induce the failure of SELEX screening (Ellington and Szostak, 1992). Above all, aptamer screening is the foundation of building aptasensors for biotoxin analysis. Currently, only dozens of aptamers have been successfully screened, which is far from enough for biotoxins. To obtain more outstanding aptamers, investigating more trustworthy aptamer screening and optimization approaches is highly demanded (Wei et al., 2023). Molecular recognition units are the core component of optical sensors, which could fundamentally determine the specificity of the method. Generally, as a kind of biological recognition molecule, an aptamer is often compared with an antibody to highlight its superior functions. Although antibodies are commonly used as recognition ligands, obtaining biotoxin antibodies based on animals is technically challenging due to their high toxicity and poor immunogenicity. Compared with antibodies, aptamers have advantages as follows: being non-immunogenic, having good chemical stability, and not involving animal ethical issues. Moreover, as aptamers are chemically synthesized, they are easy to be structurally modified for improving the application performance (McKenzie et al., 2021).

## 3 Optical aptasensors for biotoxins

**FIGURE 2:**
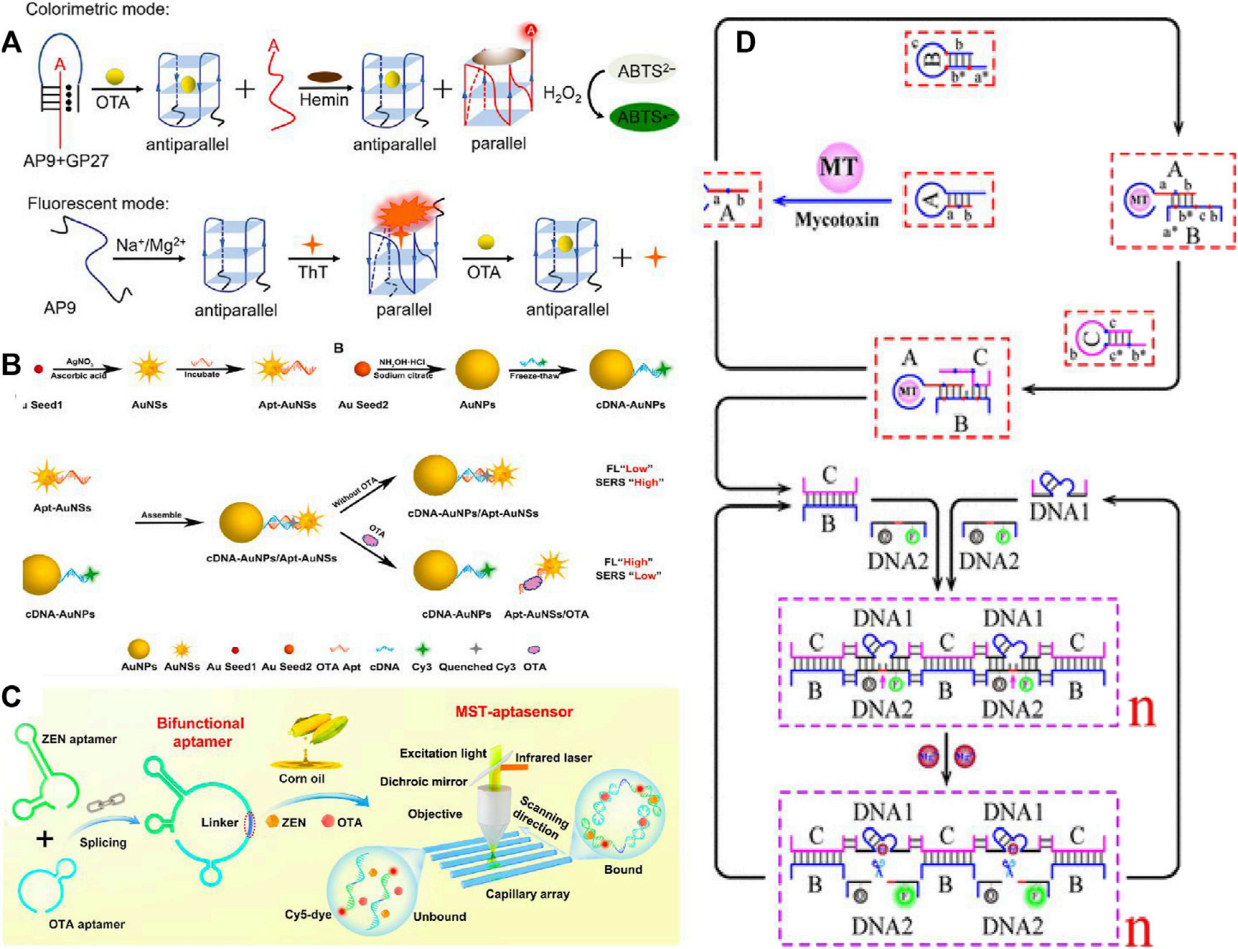
Schematic illustration of **(A)** the label-free, rapid, and sensitive detection of ochratoxin A in colorimetric and fluorescent modes by engineering DNA G-quadruplex (Balbinot et al., 2021). **(B)** FL-SERS dual-mode detection of OTA (Zhang N. et al., 2022). **(C)** Engineered bifunctional aptamer and the MST assay (Tang and Li, 2017). **(D)** Detection mechanism based on a toehold-mediated cascade catalytic assembly and supramolecular DNAzyme nanostructures ([B/C/DNA1/DNA2]n) for mycotoxin detection (Tang et al., 2024).

Optical aptasensors are always designed as portable analysis devices which use aptamers as recognition components to convert specific binding into measurable optical signals. Furthermore, benefiting from the recent advancement of nanomaterials (Niazi et al., 2022) and room-temperature nucleic acid signal amplification, these progresses push optical aptasensors of biotoxins to ultrasensitive detection levels (Li et al., 2019; Lei and Guo, 2022). Compared with existing detection methods, aptasensors do not require complex sample purifications and can complete sample detection within 30 min, which are more suitable for *in situ* detection, batch screening, and rapid diagnosis (Balbinot et al., 2021) (Figure 2).

**FIGURE 3:**
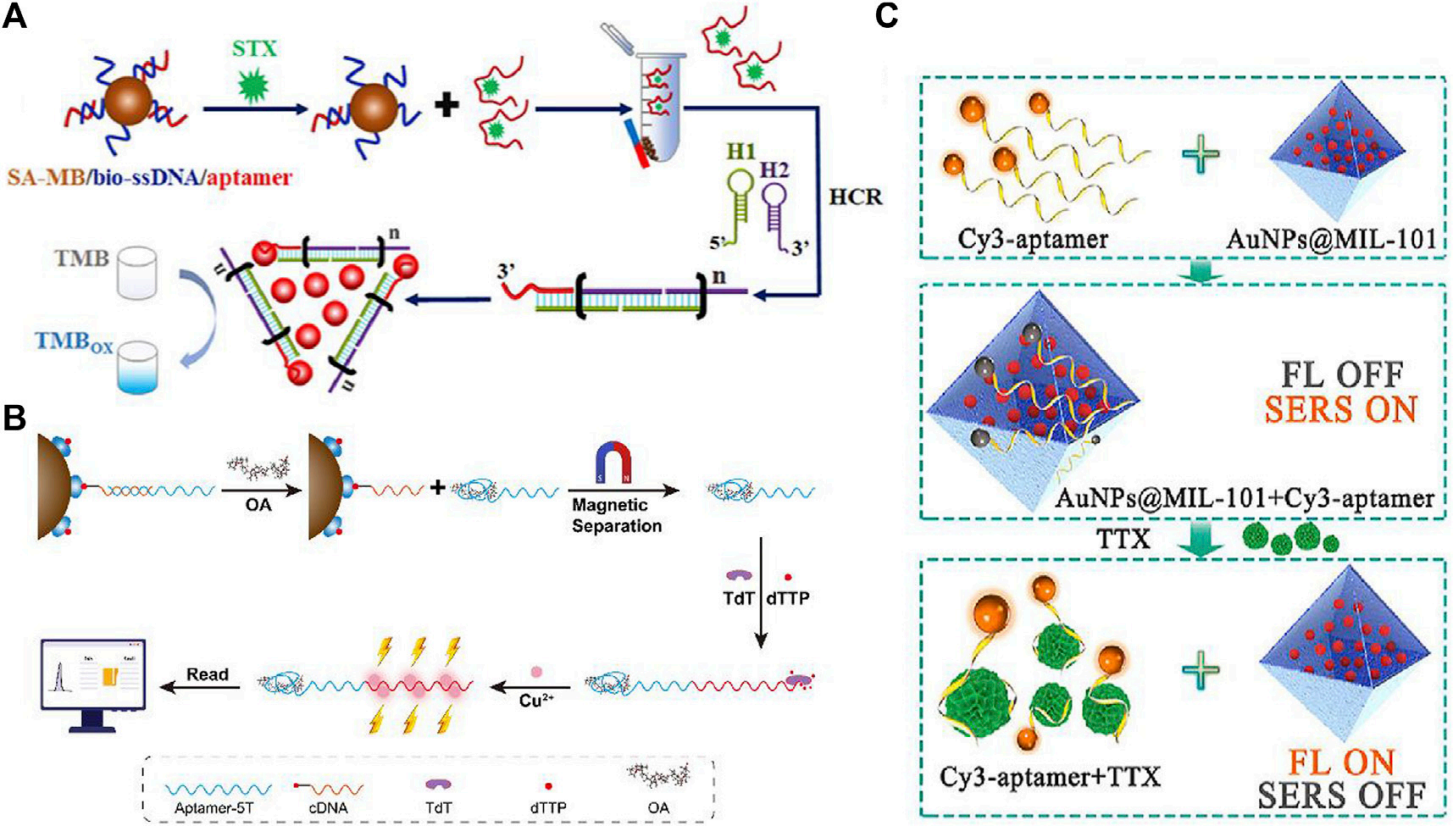
Schematic illustration of **(A)** the competitive colorimetric aptasensor transduced by hybridization chain reaction-facilitated catalysis of AuNP nanozyme for highly sensitive detection of saxitoxin (Gu et al., 2021). **(B)** Duplexed aptamer-isothermal amplification-based nucleic acid CuNP sensor for the detection of OA (Ali et al., 2014). **(C)** Fluorescence/SERS dual-mode aptasensor for analysis of TTX (Liu et al., 2019).

The exploration of biotoxin aptamers as sensing elements has made unprecedented progress in the past 3 decades. Several advanced nanomaterials have been applied for constructing various optical aptasensors for rapidly detecting biotoxins (Zhang N. et al., 2022). The colorimetric aptasensor is an instrumental independent visual sensing manner (Hizir et al., 2016; Tang and Li, 2017; Tang et al., 2024). The fluorescent aptasensor is mainly utilized for sensing biotoxins by rapidly converting target-induced aptamer conformation changes to highly sensitive fluorescent signals (Chen et al., 2017; Zhang Q. et al., 2022; Khan et al., 2023a). The SERS aptasensor, recognized as a promising analytical technique of biotoxin detection (Muhammad and Huang, 2021), possesses multiple advantages of high sensitivity, rapid reports, and non-destructiveness (Zhao X. et al., 2021). Developing outstanding SERS substrates is highly necessary for building aptasensors (Zhao P. et al., 2021). In addition to pushing the development on nanomaterial-based signal reporting units, room temperature isothermal signal amplification strategies based on complementary hybridization of nucleic acids have also been regarded as important strategies to fulfill sensing signal amplification (Gu et al., 2021; Tan et al., 2021) (Figure 3). In this aspect, enzyme-assisted nucleic acid amplification (rolling circle amplification (RCA) with the assistance of DNA polymerases) (Ali et al., 2014), a kinetics-controlled enzyme-free nucleic acid amplification technique such as hybridization chain reaction (HCR) (Bi et al., 2017) and the catalytic hairpin assembly (CHA) (Liu et al., 2019) are all novel strategies used in optical aptasensors. Furthermore, the signal amplification methods assisted by nucleases, DNAzyme, such as exonuclease I (Exo I) (Lan et al., 2020), exonuclease III (Exo III) (Wang et al., 2021), deoxyribonuclease I (DNase I) (Gu et al., 2021), and ribonuclease H (RNase H) (Gu et al., 2021) have also shown great opportunities for constructing biotoxin-specific aptasensors. Beneficial from the biodegradable properties of both double-stranded ssDNA (dssDNA) and double-stranded DNA (dsDNA) in the presence of nucleases, the biotoxins can repeatedly participate in the biological cascade reaction (BCR), thus producing signal amplification (Ren et al., 2022).

Moreover, smart synthetic DNA systems including DNA walker and the CRISPR-Cas system were also applied in fabricating aptasensors due to their programmatic behavior. Specifically, the repetitive cleavage reaction through highly directional mechanical movements was originally prelocked by the aptamer. The binding of biotoxins to the aptamer walker systems would activate this sensing system (Chen et al., 2022). For the CRISPR-Cas-based sensing system, the aptamer worked as a Cas protein activation chain. In the presence of biotoxins, the trans-cleavage activity of the Cas protein would be inhibited (Lin et al., 2022). In the nuclease-assisted signal amplification sensing systems, enzyme instability and rigorous reaction conditions might be adverse factors. Therefore, enzyme-free signal amplification aptasensors showed better durability in real applications. In this section, we described the current situation of high-performance optical aptasensors according to different types of biotoxins.

### 3.1 Optical aptasensors for mycotoxins

Mycotoxins are highly toxic secondary metabolites secreted by fungi. They are highly heat-resistant; therefore, they are hard to be removed through cooking and easily traverse into the food chain, affecting human and animal health (Eskola et al., 2020). According to the EU Rapid Alert System for Food and Feed (RASFF) statistic, mycotoxins might contaminate approximately a quarter of the world’s food and oil crops per year. Currently, agencies such as the World Health Organization and the Food Agriculture Organization have determined the permissible limit for mycotoxin contamination in food and feed to ensure food safety.

Colorimetric assay based on aggregation-induced Au/Ag NP color change, which are realized through precious regulating stability of NPs in a salt solution via an aptamer, has been widely used for designing visual mycotoxin aptasensors (Mirón-Mérida et al., 2021). In an AgNP-based colorimetric aptasensor, aflatoxin B1 (AFB1) achieved sensitive detection with an LOD of 0.09 ng/mL in food samples (Lerdsri et al., 2021). The signal amplification strategy was also widely integrated to the optical aptasensors for monitoring mycotoxins. The sensing platform was proposed with an LOD of 0.62 ng/mL, which could be utilized for AFB1 monitoring in complex sample matrices (Chen et al., 2016). Utilizing the peroxidase-like activity of AuNPs, a colorimetric aptasensor for zearalenone (ZEN) was designed using a chimeric aptamer. The aptasensor provided an LOD of 0.58 ng/mL, which could be well applied in real corn oil samples (Liu M. et al., 2022).

Fluorescent aptasensors have shown advantages of facile signal transduction, fast response, and high sensitivity, but they suffer from poor signal stability. In these designs, Förster resonance energy transfer (FRET) and the time-resolved fluorescent manner could overcome these shortcomings; thus, they have been widely utilized in the analysis of mycotoxins. Wang et al. designed a label-free aptasensor for FRET detection of trichothecenes A (T-2) toxin as low as 0.93 pg/mL. In this assay, T-2-specific aptamer functionalized using silver nanoclusters (apt-AgNCs) was synthesized as the fluorescent probe, whose fluorescence was initially quenched by MoS_2_ nanosheets (NSs). The presence of T-2 biotoxin led to the desorption of Apt-AgNCs from MoS_2_ NSs, which caused the fluorescence recovery in a target amount-dependent manner. This method showed good utility in risk assessment of T-2 toxin (Khan et al., 2018). Using a similar design, Wang et al. further synthesized a green-emitting gold nanocluster (Arg@ATT-AuNCs) as signal reporting element for the fluorescent sensing of T-2. The bioassay showed an LOD of 0.57 pg/mL with a linear range of 0.001–100 ng/mL (Khan et al., 2020).

Moreover, advanced nanomaterials have gained much attention for constructing sensors with enhanced performance that detect multiple mycotoxins simultaneously due to their creditable optical properties. Niazi et al. proposed a rapid time-resolved fluorescence (TRF)-based aptasensor for simultaneous recognition of ochratoxin A (OTA) and fumonisin B1 (FB1) using a multi-color, Ln-doped NPs (TRF-NPs) group. After method optimization, LODs of 0.019 pg/mL and 0.015 pg/mL for FB1 and OTA were achieved, respectively (Niazi et al., 2019a). In another case, a turn-on time-resolved fluorometric aptasensor is described for the simultaneous detection of ZEN, T-2, and AFB-1 in maize samples based on the multi-color, TRF-NP group, and tungsten disulfide nanosheets (WS_2_ NSs) are used as a quencher of time-resolved fluorescence. These methods showed great potentials in food safety fields (Niazi et al., 2019b). Based on a similar design, Khan et al. (2019) proposed a multicolored nanomaterial-based FRET sensing platform for the simultaneous detection of mycotoxins. The assay combined the dual-color gold nanoclusters (AuNCs) as fluorescence donors with WS_2_ NSs as a fluorescence quencher, which achieved simultaneous recognition of AFB1 and ZEN with a detection limit of 0.34 pg/mL and 0.53 pg/mL, respectively.

Hitabatuma et al. (2021) developed an aptasensor for OTA with an LOD of 0.247 pg mL^−1^. The aptamer binding to OTA induced the cDNA to hybridize with molecular beacon (MB). Following the MB stem unwinding, the FAM labeled on the MB would be far from the DABCYL. The fluorescence “turn-on” sensing mechanism is simple and fast, which exhibited specificity and sensitivity (LOD of 0.247 pg/mL) to OTA in a wheat sample. Fan et al. (2021) reported a dual-emission aptasensor based on the two fluorescent dyes: thioflavin T (ThT) and trans-2-[4’-(dimethylamino)styryl]-3-ethyl-1,3-benzothiazole (DMASEBT). The fluorescence of ThT was quenched after being inserted into DNA strands. In the working state, AFB1 would displace the bound ThT to the solution. The aptasensor was applied to the analysis of AFB1 with an LOD of 0.01 ng/mL in food samples successfully. Attributing to the development of novel nanomaterials, cobalt oxyhydroxide (CoOOH) nanosheets and graphitic carbon nitride quantum dots (gCNQDs) were used to fabricate a FRET-based aptasensor for OTA detection. This method was featured with ultra-sensitivity with an LOD as low as 201.9 pg/mL and was successfully applied to corn and barley flour (Bi et al., 2020). A sensitive fluorescent aptasensor for AFM-1 was proposed based on the time-resolved fluorescent NP as a signal probe and RCA to improve the sensitivity of the assay. The assay showed a lower detection limit (0.0194 pg/mL) than the previously reported assays (Niazi et al., 2020). In addition, CRISPR-Cas-assisted fluorescent aptasensors were constructed to analyze mycotoxins. Mao et al. (2022) applied CRISPR/Cas12a in designing an aptasensor for OTA detection. In the sensing process, an activated cDNA strand was first released from the sensors in the presence of OTA. The cDNA was subsequently hybridized with crRNA to cut UCNP-DNA linked on the Fe_3_O_4_ NPs, and then the fluorescence signal was turned on. This method was very sensitive, with an LOD of 0.83 ng/mL. The CRISPR/Cas12a-based sensing strategy had a great practical application prospect for various targets (Mao et al., 2022). SERS are well known as an attractive analytical tool with advantages of rapid and on-site ultrasensitive detection. SERS-based aptasensors are well developed as a promising tool for biotoxins. Constructing outstanding SERS substrates and SERS tags with superior properties is the key technology in this field. Chen et al. (2021) proposed a universal aptasensor for the analysis of ZEN based on Fe_3_O_4_@Au NPs and Au@Ag core-shell NPs, which could perform well in food samples with an LOD of 1.0 ng/L. A ratiometric SERS aptasensor for AFB1 was constructed based on hybrid nanomaterial, that is, Ti_3_C_2_T_x_ MXene-loaded AuNP dimers. Assembled AuNP dimers contained a rich SERS “hot spot,” which provided a strong SERS signal. MXenes nanosheet functioned as a support to the aptamer-modified AuNP dimers for achieving steady Raman signal. The presence of AFB1 could competitively bind to the aptamer, thus pushing AuNP dimers to be separated from MXenes NSs. This recognition processing causes the SERS intensity to decay with the increase of AFB1. The aptasensor showed an LOD as low as 0.6 pg/mL and could be well used in peanut samples (Wu et al., 2022).

To further improve the sensing reliability, dual-mode optical aptasensors have been developed for mycotoxin detection. Based on the feature of the G-quadruplex structure, He et al. (2022) developed a label-free and dual-mode aptasensor for OT, which contained both G-quadruplex/hemin DNAzym as the catalytic colorimetric unit and G-quadruplex ThT as the fluorescence reporting unit. Following the colorimetric manner, a DNA triplehelix switch was composed of aptamer and G-rich sequences. The binding of OTA resulted in the separation of the triple-helix, which subsequently released the ssDNA to bind hemin and stimulate color change. Under the fluorescent mode, the aptamer would combine with ThT to produce a strong signal. Beneficially from the structure flexibility of ssDNA, the G-quadruplex DNA assembly was rationally engineered to achieve dual-mode sensing for biotoxins. For instance, two kinds of NPs including AuNSs and AuNPs were modified with the aptamer and Cy3-modified cDNA, respectively. In the presence of OTA, the hybridization of aptamer and cDNA was broken, causing the disassembly of AuNSs and AuNPs. This process turned on the fluorescenc Cy3, but the SERS signal from Cy3 was decreased, achieving a dual-mode OTA ratio analysis. The aptasensor had good practicality in the analysis of coffee and wine samples (Wang et al., 2022).

Moreover, simultaneously detecting aptasensors have also been developed due to their outstanding time- and cost-saving features as well as fast and high-throughput testing. Optical aptasensors can also be constructed to simultaneously monitor multiple mycotoxins. Yang et al. (2021) designed a dual-targeted aptamer, which is the tandem of ZEN aptamer and OTA aptamer *via* a poly-T linker. Using this novel aptamer, simultaneous determination of ZEN and OTA as low as 0.12 nM was achieved based on the microscale thermophoresis in corn oil samples. Xiong et al. (2021) designed a dual DNA tweezer nanomachine that was originally inhibited by the anti-AFB1 aptamer and anti-OTA aptamer; both of them were labeled FAM and Cy5 fluorophores, respectively. The fluorescence was turned on in the presence of targets. The methods also showed good performance in cereals with LODs of 0.035 ng/mL for AFB1 and 0.1 ng/mL for OTA. Signal amplification based on CHA- and DNAzyme-cascaded hydrolysis reaction has also been applied for simultaneously sensing multiple mycotoxins. Several concatenated logic gates were used in the biosensors due to the versatility of these methods (Pan et al., 2022), which demonstrated that the multifunctional logic system had great potential for constructing biosensors for multiple mycotoxins. SERS aptasensors can also be designed to analyze multiple mycotoxins in one test. Song et al. (2022) developed an aptasensor based on diverse SERS tags and AuNP-modified 3D silica photonic crystal microsphere (SPCM) array with low LODs of 0.36 pg/mL for AFB1 and 0.034 pg/mL for OTA. Dual-mode aptasensors can also be developed for the simultaneous detection of multiple mycotoxins. For instance, based on the FRET and SERS manners, Wu et al. (2020) proposed an aptasensor for the simultaneous analysis of ZEN, FB1, and OTA. A long cDNA-labeled AuNP was designed to hybridize with the ZEN aptamer-labeled UCNP, the FB1 aptamer-labeled AuNP, and the OTA aptamer-labeled Cy5 simultaneously. In the presence of targets, the fluorescence signal of the UCNP or Cy5 increased, whereas the SERS signal of the AuNP decreased. Au nano-hybrid structures have emerged as important sensing materials, which could be synthesized to improve sensitivity and specificity. In Khan’s work, a fluorescence-labeled silica shell with Au NPs as the core was synthesized as a FL/SERS dual-mode nanoprobe for T-2 toxin. When exposed to T-2 toxin, the sensing system showed the concentration-dependent restoration of FL with the reduction of the SERS signal with the LOD of 85 p.m. Compared with ELISA, this method presented superior performance in wheat and maize (Khan et al., 2023b).

### 3.2 Optical aptasensor for marine toxins

Marine toxins, mainly generated by algae or phytoplankton during harmful algal blooms, are generally highly toxic. As they are continuously released in the environment, marine toxins are easily accumulated in aquatic and marine organisms such as mollusks and fishes through the food chain, finally posing a serious health threat to humans *via* consuming toxin-contaminated seafood (Wang et al., 2024). Thousands of marine toxins poisoning cases have been reported in the 21st century (Qiang et al., 2020). Marine toxin-contaminated food could lead to foodborne diseases such as food poisoning, diarrhea, indigestion, and neurotoxicity, even at low doses. In the United States, more than 3,000 people die owing to foodborne diseases annually. Therefore, it is highly necessary to design rapid, high-throughput, and cost-effective methods for the detection of multiple food contaminants. Thus, rapid detection of marine toxins in food is also an urgent task for health and safety. In this part, we summed up the optical aptasensors for typical marine toxins such as STX, OA, TTX, PTX, BTX, and DA (Nicolas et al., 2017).

Qiang et al. (2020) reported a colorimetric aptasensor based on salt-induced AuNP aggregation for quantitative analysis of STX at a concentration as low as 10 fM. By combining Au nanozymes and aptamer-triggered HCR, Li et al. proposed a colorimetric aptasensor for STX. By catalyzing the oxidation reaction of TMB-H_2_O_2_, an aptasensor with an LOD of 42.46 p.m. was achieved in real scallop samples (Zhao Y. et al., 2021). Fluorescent aptasensors have also been well developed for recognizing highly sensitive marine toxins. Xie et al. (2021) created an aptasensor for MC-LR analysis using FAM-labeled aptamers/AuNPs and DNase I, thus realizing fluorescence signal amplification. Shan et al. (2022) proposed a highly sensitive aptasensor for OA in seafood. In the presence of OA, they could bind with aptamers and produce long sequences with a poly(thymine) tail. The poly(thymine) tail becomes the copper nucleation sit. The fluorescence intensity derived from Cu nanoclusters could be in line with the OA concentrations. The aptasensor could achieve ultra-high sensitive detection of OA with an LOD of 1.1 pg/mL. Cheng reported an SERS-based aptasensor for STX for the first time. In the presence of STX, the STX would bind with aptamer, thus inducing tag molecules far from SERS substrate and the consequent SERS signal attenuation. This SERS aptasensor could be applied in shellfish samples with an LOD of 3.51 ng/mL (Cheng et al., 2019).

Introducing dual-mode sensing could improve the sensing performance of marine toxins in foods. Liu S. et al. (2022) demonstrated a fluorescence/SERS dual-mode aptasensor for the analysis of TTX. The Cy3 labeled aptamers were bound on the surface of novel AuNPs/MOF nanohybrids (MIL-101), which demonstrated fluorescence quenching and SERS enhancement. The presence of TTX would result in the fluorescence signal “turn on” and the SERS signal damping. This method showed distinguished sensitivity with an LOD of 6 pg/mL and demonstrated excellent practicability for screening TTX in puffer fish and clam. There were also novel aptasensors fabricated for sensing multiple typical marine toxins in one test. For example, Li et al. (2022) constructed a novel aptasensor for achieving simultaneous detection of three diarrheic shellfish toxins, including OA, DTX-1, and DTX-2. By integrating the TF-DSP aptamer with AuNPs@Fe^2+^ nanozyme activity, the assay showed good performance in real seafood samples.

### 3.3 Optical aptasensor of phytotoxins and bacterial toxins

**FIGURE 4:**
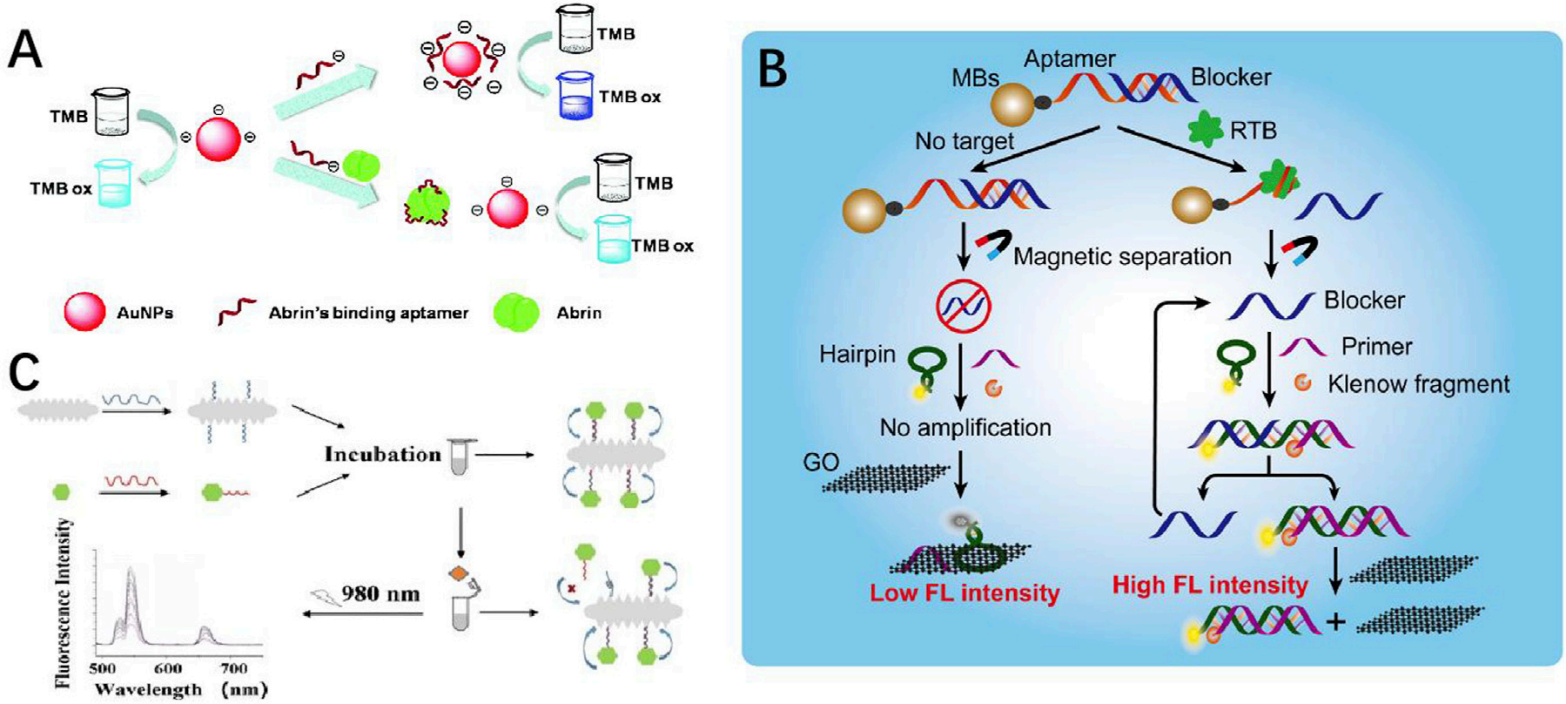
**(A)** Mechanism of the aptamer-based colorimetric biosensor for abrin (Ren et al., 2022). **(B)** Proposed ricin B-chain detection strategy (Chen et al., 2022). **(C)** Aptasensor based on AuNR@Pt-UCNPs for sensitive detection of SEB (Chen et al., 2016).

Phytotoxins are naturally toxic plant-derived chemicals or proteins including siteloids, pyrrolizidine alkaloids, and lectins, and they could internalize into human cells and cause serious harm by inhibiting protein synthesis. Some of them are highly toxic to animals and humans if they contaminate food. Recent advancements in optical aptasensors have shown the availability for detection and monitoring (Günthardt et al., 2018). The reported aptamers for phytotoxins are mainly in ricin and abrin. Based on the unique peroxidase-like activity of aptamer-modified AuNPs, Hu et al. (2015) developed a simple colorimetric aptasensor for abrin (Figure 4). This method showed an LOD as low as 0.05 nM. Li et al. (2017) developed a novel strategy for analysis of ricin B-chain (RTB) based on isothermal strand-displacement polymerase reaction (ISDPR). In this design, a short blocker ssDNA was originally hybridized with the aptamer. In the presence of ricin, the blocker ssDNA was released and then hybridized with florescence-labeled hairpin probes to activate strong fluorescence. This technique could be applied to detect the RTB as well as the entire ricin toxin in the juice with an LOD of 0.6 mg/mL. To improve the performance of the “kinetic competition” aptasensor in complex matrices, Qi et al. proposed a ratiometric “kinetic competition” aptasensor using a dual fluorescence-labeled probe for RTA detection (Qi et al., 2020). The method was verified as a feasible method for RTA in sucrose, yeast, and baking soda powder samples.

Bacterial toxins are a type of chemical derived from *Clostridium*, *Salmonella*, *Staphylococcus*, and *Listeria* pathogens that can invade host cells to cause foodborne infections in humans (Braun et al., 1994). Bacterial contamination has shown diverse causes, such as through unwashed hands, being present in raw milk or meat, and through contaminated water. By utilizing a similar aptamer-mediated gold nanoparticle aggregation mechanism, a rapid and easy colorimetric sensing was achieved for SEB using its aptamer and AuNPs in milk samples with LODs of 50 ng/mL visually and 0.5 ng/mL (Mondal et al., 2018). In addition, Wu et al. developed a fluorescent aptasensor for the analysis of SEB based on the aptamer-functionalized AuNR@Pt module and the cDNA-immobilized UCNP module. In another case, a fluorescent aptasensor for the analysis of SEA was designed by combining the aptamer functionalized AgNCs unit with the polypyrrole nanoparticles (PPyNPs). Aptamers could non-covalently bind onto the PPyNP, making the fluorescence of AgNCs turn on. The binding of SEA with the aptamer resulted in the release of AgNCs from the PPyNPs; therefore, the fluorescence was turned on under the stimulus of the target. In this method, an LOD of 0.3393 ng/mL was achieved in milk samples (Zhang et al., 2020).

### 3.4 Current challenges of optical aptasensors for biotoxins

In the past 3 decades, we have witnessed significant advancements in analytical techniques to manage the food safety problem of biotoxin contamination. UPLC–MS-based biotoxin analysis, including facilities, sophisticated analytical instruments, reagents, and logistics, made them unsuitable for daily monitoring. Currently, rich kinds of optical aptasensors were designed and they further demonstrated their real applications for biotoxins, including a variety of advanced analytical techniques and ideas, which could reduce the time and cost requirements. However, there are still some challenges that should be considered, such as authority, stability, and reliability, which make on-field rapid detection difficult. Here, we listed some significant challenges for rapid screening of biotoxins that are commonly encountered:

First, sample preparation in optical sensing is the inescapable critical step in real application. It was revealed that nearly 30% of the method variability originated from sample preparation. By paying attention to the key factors of sample preparation (size reduction, sampling size, and uniformity), the quality of rapid sensing results could be improved significantly. Considering the highly harmful nature of the biotoxins, the acceptable concentration range of different toxins varied in the same samples. Therefore, the sensing method should fully consider real needs. Third, food matrices are highly complex, which go through different processing processes, thus posing huge interferences for precise detection.

Rapid screening of biotoxins in foods shows huge market prospects in the future. Taking the mycotoxin testing market as an example, the testing market is set to reach a cumulative annual growth rate (CAGR) of 7.8%, which corresponds to a market of $1.4 billion by 2026 (EMR, 2022). As it indicates, food safety testing is an accelerated social need. In the light of this situation, the optical aptasensor should be further modified for higher precision while achieving the detection of multiple toxins simultaneously with high sensitivity and cost efficiency.
